# Long-Term Course of Failed Back Surgery Syndrome (FBSS) Patients Receiving Integrative Korean Medicine Treatment: A 1 Year Prospective Observational Multicenter Study

**DOI:** 10.1371/journal.pone.0170972

**Published:** 2017-01-27

**Authors:** Jinho Lee, Joon-Shik Shin, Yoon Jae Lee, Me-riong Kim, Areum Choi, Jun-Hwan Lee, Kyung-Min Shin, Byung-Cheul Shin, Jae-Heung Cho, In-Hyuk Ha

**Affiliations:** 1 Jaseng Spine and Joint Research Institute, Jaseng Medical Foundation, Seoul, Republic of Korea; 2 Clinical Research Division, Korea Institute of Oriental Medicine, Daejeon, Republic of Korea; 3 University of Science & Technology (UST), Korean Medicine Life Science, Campus of Korea Institute of Oriental Medicine, Daejeon, Republic of Korea; 4 Division of Clinical Medicine, School of Korean Medicine, Pusan National University, Yangsan, Republic of Korea; 5 Department of Korean Rehabilitation Medicine, Kyung Hee University, Seoul, Republic of Korea; Institute of medical research and medicinal plant studies, CAMEROON

## Abstract

**Background:**

With increase of spine surgeries, failed back surgery syndrome (FBSS) prevalence is also rising. While complementary and alternative medicine (CAM) is commonly used for low back pain (LBP), there are no studies reporting use of integrative Korean medicine in FBSS patients.

**Methods:**

Patients with pain continuing after back surgery or recurring within 1 year and visual analogue scale (VAS) of LBP or leg pain of ≥6 (total n = 120) were recruited at 2 hospital sites from November 2011 to September 2014. Weekly sessions of integrative Korean medicine treatment were conducted for 16 weeks (herbal medicine, acupuncture/electroacupuncture, pharmacopuncture/bee venom pharmacopuncture, and Chuna manual therapy) with additional follow-ups at 24 weeks and 1 year. Outcome measures included VAS of LBP and leg pain (primary outcome), Oswestry Disability Index (ODI), Short-Form 36 (SF-36), medical use, and patient global impression of change (PGIC).

**Results:**

VAS of LBP and leg pain improved at 6 months (LBP from 6.1±2.0 at baseline to 2.9±2.3; and leg pain from 5.4±2.6 to 2.4±2.5, respectively). Eighty patients (66.7%) showed improvement of 50% or more in main pain of LBP or leg pain from baseline. Disability and quality of life also improved at 6 months (ODI from 41.3±12.3 at baseline to 23.6±13.6; and SF-36 from 42.8±14.5 to 62.7±16.8). At 1 year follow-up, conventional medical management use decreased, improvement in pain and disability was maintained, and 79.2% reported improvement of PGIC.

**Conclusions:**

Despite limitations as an observational study, integrative Korean medicine treatment showed positive results in pain, function, and quality of life of FBSS patients.

## Introduction

Low back pain (LBP) and radiating leg pain have heavier impact on time off work and disability than any other medical condition [1]. Although natural progress of LBP is known to be favorable, surgery rates are high. It was reported that 317,000 lumbar surgeries were conducted in the U.S. in 1997 [2], and numbers climbed to 1 million in 2002 [3]. Also, despite lack of clear evidence for spinal fusion, rates have increased 220% between 1990 and 2000 [3,4]. Operation results are not always successful, and cases where LBP and/or leg pain persists or relapses are commonly referred to as failed back surgery syndrome (FBSS) [5,6].

Incidence of FBSS can be roughly inferred from the proportion with unsuccessful outcomes in lumbar surgery-related trials, which is estimated at 4–50%, and incidence in the general population is put at 0.02% in lower estimate and 2% in higher estimation [7–11]. A recent systematic review reports that quality of life in FBSS patients is poor, and that it is lower compared not only to neuropathic pain disorders (diabetic polyneuropathy), but also other chronic diseases (stroke, heart failure) [12].

In treatment of FBSS patients, multidisciplinary approaches have been purported to be most effective [13], and recently some clinical studies appear to have embraced this trend, comparing spinal cord stimulation (SCS) with an active control of optimal medical management (OMM), i.e. customized conventional medical management methods including medication, injection, epidural steroid injection (ESI), physical therapy, and cognitive treatment [14–16].

A considerable portion of patients who found conventional medical management ineffective are turning to complementary and alternative medicine (CAM) treatment for LBP, with one survey stating that 43% of peripheral neuropathy patients use CAM [17]. Integrative Korean medicine treatment is currently utilized for various LBP-related disorders in Korea, which is in line with the “Korean Medicine Clinical Practice Guideline for Lumbar Herniated Intervertebral Disc in Adults” recommendations for multimodal over singular treatment on the basis of higher effectiveness [18]. According to a survey conducted mainly in Korean medicine doctors (KMDs) employed at spine-specialty Korean medicine hospitals (hospitals accredited by the Korean Ministry of Health & Welfare to provide advanced medical care for a specialty/disease), many lumbar disc herniation patients were receiving a multimodal treatment approach to LBP consisting of acupuncture, herbal medicine, pharmacopuncture/bee venom pharmacopuncture, and Chuna manual therapy [19].

However, there are no studies on the effect of integrative Korean medicine treatment in FBSS patients, and in light of the fact that many patients are selecting CAM or Korean medicine for LBP treatment, related research is in urgent demand. The authors have previously studied the effect of integrative Korean medicine treatment (acupuncture, herbal medicine, pharmacopuncture/bee venom pharmacopuncture, and Chuna manual therapy) for lumbar disc herniation patients in a prospective observational study, and reported the 6 month [20], 3 year [21], and 5 year results [22]. The present study was conducted with the aim of investigating if and how integrative Korean medicine treatment affects pain, function, quality of life, current medical use, and safety in FBSS patients.

## Materials and Methods

This study is a prospective, multicenter, observational study. Patients with unremitting or recurrent LBP or leg pain after lumbar surgery were recruited at 2 sites of Jaseng Hospital of Korean Medicine (Gangnam branch in Seoul, and Bucheon branch) in Korea from November 2011 to September 2014. The majority of participants were not recruited by advertisements but were patients approached upon visiting the hospital for treatment. Jaseng Hospital of Korean Medicine is a spine specialty hospital certified by the Korean Minister of Health and Welfare that pursues an integrative treatment model of conventional and Korean medicine based on conventional diagnostic technology in diagnosis and Korean medicine treatment for the main treatment modality [23,24]. This study is a report of the 1 year observation results of effect and safety of integrative Korean medicine treatment in FBSS patients.

**ClinicalTrials.gov Identifier:** NCT01701804

### Inclusion criteria

1. Patients with LBP or leg pain that did not improve with spine surgery or recurred within 1 year
2. Patients with current LBP or leg pain with duration of at least 3 weeks
3. Patients with LBP or leg pain of 60mm or higher on visual analogue scale (VAS) scale
4. Patients aged 18 years or older, and 60 years or younger
5. Patients who are able to take magnetic resonance imaging (MRI) scans and have given consent to taking lumbar MRIs
6. Patients who are capable of free expression of opinion and have given voluntary written informed consent to participate
7. Patients who have agreed to refrain from receiving other pain treatments relating to spinal disorders during the clinical study participation period

### Exclusion criteria

1. Patients with diagnosis of specific serious diseases which are possible causes of spinal pain: e.g. malignant tumors, vertebral fractures, spinal infection, inflammatory spondylitis, cauda equina syndrome
2. Patients with progressive neurological deficit or severe concurrent neurologic symptoms
3. Patients with pain from non-spinal causes and/or soft tissue problems: e.g. tumors, fibromyalgia, rheumatoid arthritis, gout
4. Patients with other chronic diseases which could potentially influence interpretation of treatment effect or results: cardiovascular disease, renal disease, diabetic neuropathy, dementia, epilepsy
5. Patients unsuitable for acupuncture, pharmacopuncture or bee venom pharmacopuncture, or at risk of acupuncture-associated safety complications: e.g. patients with hemorrhagic disease (clotting disorders), patients under anticoagulant treatment, severe diabetes patients with risk of infection, serious cardiovascular disease patients, patients with history of severe allergic reaction following bee venom pharmacopuncture or bee sting
6. Patients currently taking corticosteroids, immuno-suppressant drugs, psychiatric medicine, or other medications deemed unsuitable by the researcher
7. Patients in pregnancy or planning pregnancy
8. Patients deemed unsuitable for clinical trial by the researcher upon grounds of diagnostic imaging results

All patients received X-ray and MRI tests before study participation, and spine surgery segment and method (e.g. fusion, laminectomy) were confirmed by radiology specialists and KMDs. However, the definition of spine surgery in this study also included microscopic spine surgeries difficult to discern through radiologic imaging (e.g. discectomy without laminectomy).

### Interventions

Integrative Korean medicine treatment was conducted in accordance with patient pain level and state. Treatment frequency was once a week for 16 weeks, and additional treatment sessions were allowed with regard to patient state. Participants were asked to refrain from receiving treatments for LBP or leg pain other than the integrative Korean medicine treatment (herbal medicine, acupuncture, electroacupuncture, Chuna manual therapy, pharmacopuncture and bee venom pharmacopuncture) or rescue medicine (nonsteroidal anti-inflammatory drugs (NSAIDs)) stated in the protocol for 16 weeks and discontinued any other LBP/leg pain-related medication. All additional treatment types and frequency was recorded. All interventions were performed by KMDs who had completed 6 years of undergraduate education and 4 years of Korean medicine specialist training courses certified by the Korean Ministry of Health and Welfare.

#### Integrative Korean medicine treatment

1. Herbal medicine: Chungpa-jun [25–28] is an herbal medicine decocted from *Ostericum koreanum*, *Eucommia ulmoides*, *Acanthopanax sessiliflorus*, *Achyranthes japonica*, *Psoralea corylifolia*, *Saposhnikovia divaricata*, *Cibotium barometz*, *Lycium chinense*, *Boschniakia rossica*, *Cuscuta chinensis*, *Glycine max*, and *Atractylodes japonica* (120ml). Standard dosage was twice a day.
2. Chuna manual therapy [29]: Chuna manual therapy is a form of Korean spinal manipulation and was administered for 5–10 minutes. Chuna manual therapy exercises high-velocity low-amplitude thrusts slightly beyond the passive range of motion (ROM) to spinal joints, and manual force to joints for spinal mobilization without thrusts within the passive ROM.
3. Acupuncture treatment: Acupuncture was performed using 0.30X40 mm disposable sterile acupuncture needles (Dongbang Acupuncture, Inc., Korea). Needles were retained for about 15 minutes per session, and electroacupuncture was applied as needed during retention of needles.
4. Bee venom pharmacopuncture: Sterilized bee venom pharmacopuncture solution was prepared through aseptic processing of 1g of dried bee venom dissolved in 10,000cc of water for pharmacopuncture injection per session applying 0.1cc at acupuncture points using 30-gauge 1cc injection needles to within a total amount of 1cc. Bee venom pharmacopuncture was administered after checking skin test results, and if positive, an alternative pharmacopuncture type was used.

The acupuncture points used in acupuncture and bee venom pharmacopuncture were generally selected from 6 common acupuncture points for LBP treatment (bilateral BL23, BL24, BL25) and Hyeopcheok points (Huatuo Jiaji, EXB2) as standard, and additional acupuncture points conforming to syndrome differentiation following Korean medicine theory were allowed. The total number of acupuncture points for acupuncture and bee venom pharmacopuncture was limited to 10–20 points.

#### Rescue medicine

NSAIDs were permitted as concomitant medication over the 16 week intervention period in patients whose pain control was insufficient with integrative Korean medicine treatment. Subjects could take up to 3 tablets a day, taking 1 tablet at 8 hour intervals.

### Outcome measures

Primary outcome measures were VAS of LBP and leg pain, which uses a 10cm horizontal line with each end defined as no pain and most severe pain imaginable, respectively, and patients were instructed to mark level of current average pain on the line [30,31].

Secondary outcome measures included Oswestry Disability Index (ODI) to assess functional status of patients [32], and the validated Korean version of ODI was used [33]. The Korean version of Short-Form 36 (SF-36) which evaluates physical functioning, role-physical, bodily pain, general health, vitality, social functioning, role-emotional and mental health [34] was used to assess quality of life [35]. The 5-leveled patient global impression of change (PGIC) was employed to evaluate current state in patients (completely improved, distinctly improved, improved, no significant difference, and worsened).

Type and frequency of medical use were also assessed. Medication history (oral, and injection type) regarding LBP and leg pain for the 2 months prior to study enrollment and all treatments other than that specified in the 16 week protocol received during the interventional period were investigated. All forms of medical use were assessed over the follow-up period of 1 year. Adverse events were investigated by assessing causal relationship with current treatment (World Health Organization-The Uppsala Monitoring Centre (WHO-UMC) causality scale) [36] and severity (Spilker's AE classification) [37], discerning serious adverse events, and inspecting safety measures, corrective treatment and outcome through patient interviews. Blood tests were conducted for safety evaluation, and involved white blood cell (WBC) count, hepatic function test, renal function test, erythrocyte sedimentation rate (ESR), and C-reactive protein (CRP).

Outcome assessments of VAS, adverse events, and medical use were performed at baseline, 2, 4, 6, 8, 10, 12, 14, 16, 20, and 24 weeks, ODI and SF-36 at baseline, 4, 8, 12, 16, 20, and 24 weeks, and PGIC face-to-face at 16, and 24 weeks. Blood was drawn for testing at baseline, 8, and 16 weeks and additional follow-up tests were conducted if considered necessary. The 1 year follow-up assessment was conducted by phone and assessed numeric rating scale (NRS), ODI, medical usage, and PGIC.

### Statistical analysis

Intention-to-treat (ITT) was performed as primary analysis for all outcomes in all patients (n = 120) who had undergone at least 1 follow-up session, and missing values were handled by last observation carried forward (LOCF). Continuous variables are expressed as mean±standard deviation (SD) and category variables as n (%), and sociodemographic characteristics, history, and medical use (medication and interventions) of study participants were investigated. Each timepoint measurement and baseline of VAS of LBP and VAS of radiating leg pain were compared to assess primary outcome variables, and difference between timepoints (baseline—each timepoint) was analyzed using paired t-test after checking for normal distribution. Analyses were performed for secondary outcome measures ODI and SF-36 in the same manner. The number of participants with decrease of 50% or more in VAS of LBP and leg pain from baseline at each timepoint was also tallied, and the following subgroups were analyzed for trends by timepoint (time by treatment interactions) using repeated measures analysis of variance.

- Onset of pain post-surgery: Relapse (initial alleviation of pain after spine surgery and recurrence within 1 year) vs. Continuation (continuous pain persisting after spine surgery)
- Duration of pain: Pain<6 months vs. Pain≥6 months
- Main site of pain (higher VAS out of LBP and leg pain at baseline): LBP vs. Leg pain
- Sex: Men vs. Women

All statistical analyses used SAS statistical package version 9.1.3 (SAS Institute, Cary, NC, USA), and significance level was set at p<0.05.

### Ethics statement

The protocol was approved by the Institutional Review Board (IRB) of Jaseng Hospital of Korean Medicine (ClinicalTrials.gov Identifier: NCT01701804).

## Results

**Fig 1:**
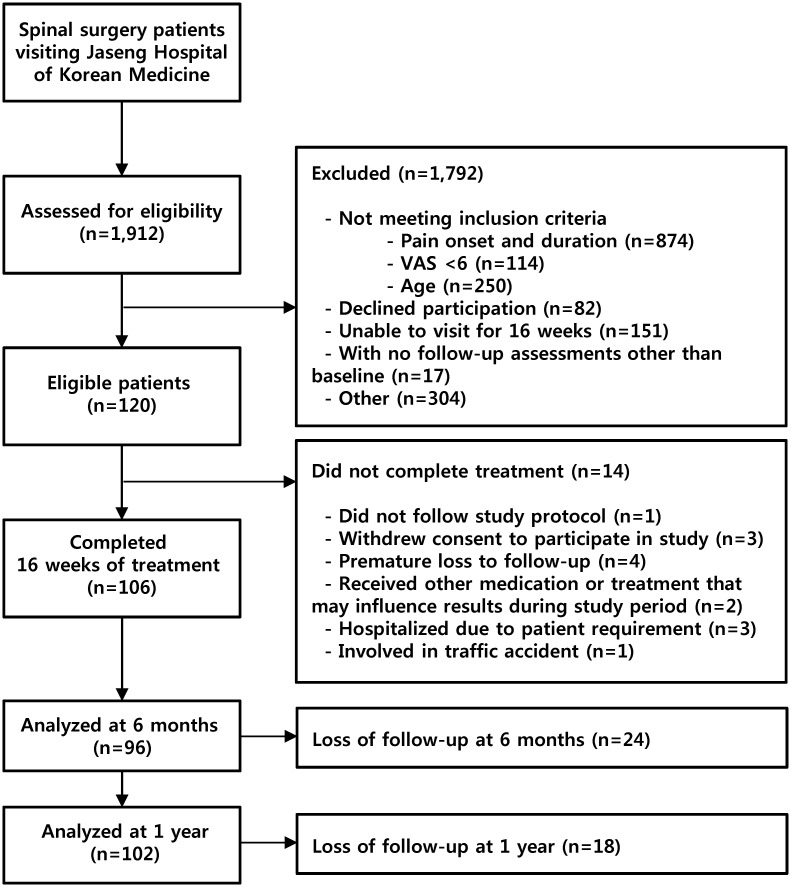
Flow diagram of the study.

A total 1,912 patients with history of spine surgery were screened, and the greater majority was excluded from reasons of pain onset, duration or pain level not meeting inclusion criteria. A total 120 patients were enrolled, of which 106 patients (88%) completed the predetermined 16 week integrative Korean medicine treatment program, and 96 patients (80%) attended the 6 month follow-up visit. The 1 year follow-up was conducted by phone in a total 102 patients (85%) out of the initially enrolled 120 patients including drop-out patients (Fig 1).

The average age of patients was 41.9±11.7 years, and 72 patients (60%) were male. Ninety-one patients (66.4%) reported pain persisting after lumbar surgery, and the rest were patients whose pain had recurred within a year of surgery after a pain-free period. Average duration of pain was 35.3±53.1 months. Ninety-two patients (77%) had undergone 1 lumbar operation, and surgical history was not evident radiologically in 19 patients (no laminectomy, fusion, or fibrosis verified on imaging). Thirty patients (25%) had received recommendations for re-surgery, and a high proportion of 101 patients (84%) replied that their prior surgery was dissatisfactory regarding satisfaction of previous surgery (Table 1).

**Table 1:** Patient characteristics at baseline (n = 120).

Variable	n (%) / mean±SD	Variable	n (%)
Age	41.9±11.7	Number of spine surgeries	
Sex		1	92 (76.7)
Male	72 (60.0)	2	21 (17.5)
Pain relapse vs. Continuation		3	7 (5.8)
Relapse^a^	41 (34.2)	Recommendation for resurgery	
Continuation^b^	79 (65.8)	No	90 (75.0)
Pain duration (month)	35.3±53.1	Yes	30 (25.0)
Suspected cause of current pain		Laminectomy^c^	
During usual activities	99 (82.5)	1 area	78 (65.0)
Injury	16 (13.3)	2 areas	20 (16.7)
Other	5 (4.2)	3 areas	1 (0.8)
Comorbidities		Fusion^c^	
Hypertension	15 (12.5)	1 area	6 (5.0)
Hyperlipidemia	8 (6.7)	2 ares	2 (1.7)
Liver-related comorbidity(e.g. fatty liver, hepatitis B)	6 (5.0)	3 areas	1 (0.8)
Diabetes	4 (3.3)	Fibrosis^c^	
Other	7 (5.8)	1 area	22 (18.3)
History		Disc protrusion^c^	
Other spine or joint surgery	18 (15)	1 area	39 (32.5)
Gynecology-related tumor	3 (2.5)	2 or more areas	18 (15.0)
Musculoskeletal disease	3 (2.5)	Disc extrusion^c^	
Other	8 (6.7)	1 area	43 (35.8)
Alcohol intake		2 areas	4 (3.3)
No	23 (19.2)	Central spinal stenosis^c^	
Yes	97 (80.8)	1 area	5 (4.2)
Smoking status		Foraminal spinal stenosis^c^	
Non-smoker	61 (50.8)	1 area	13 (10.8)
Past smoker	24 (20.0)	2 or more areas	9 (7.5)
Current smoker	35 (29.2)		
Regular exercise		Satisfaction level of previous surgery (patient survey)	
No	50 (41.7)	Satisfied	19 (15.8)
Yes	70 (58.3)	Dissatisfied	101 (84.2)
Common working position^d^		Level of relationship between previous surgery and current pain (patient survey)	
Sedentary (seated)	79 (65.8)	Associated	82 (68.3)
Mobile (standing or walking)	25 (20.8)	Moderate	22 (18.3)
Other	17 (14.2)	Not associated	16 (13.3)

^a^ Initial alleviation of pain after spine surgery and recurrence within 1 year

^b^ Pain persisting after spine surgery

^c^ MRI readings by radiology specialists at baseline

^d^ Multiple response allowed. One patient answered affirmatively to both seated and standing or walking.

In primary outcomes of LBP and leg pain, LBP displayed a mean change of 3.2 (95% CI: 2.7–3.6) at 24 weeks compared to baseline, and that of leg pain was 3.0 (95% CI 2.5, 3.5). Also, a total 80 patients (66.7%) reported decrease in VAS of 50% or greater at main site of pain compared to baseline (site with more pain at baseline out of LBP and leg pain). Regarding mean change of function and quality of life scores, ODI improved 17.7 points (95% CI: 14.8, 20.5) at 24 weeks compared to baseline, and SF-36 showed a 19.9 point (95% CI: 16.9–22.9) increase (Table 2).

**Table 2:** Pain, function, and quality of life by timepoint (n = 120; ITT analysis).

Index			Baseline	4 weeks	8 weeks	12 weeks	16 weeks	24 weeks	48 weeks
**VAS (cm)**	**LBP**	mean±SD	6.1±2.0	4.8±2.0	4.2±2.1	3.7±2.1	3.2±2.2	2.9±2.3	3.3±2.3*
		diff	-	1.3 (1.0, 1.6)	1.9 (1.5, 2.3)	2.4 (2.0, 2.9)	2.9 (2.5, 3.4)	3.2 (2.7, 3.6)	-
	**Leg pain**	mean±SD	5.4±2.6	4.5±2.5	3.7±2.5	3.1±2.4	2.7±2.5	2.4±2.5	1.7±2.4*
		diff	-	0.9 (0.5, 1.2)	1.7 (1.3, 2.1)	2.3 (1.8, 2.7)	2.7 (2.2, 3.1)	3.0 (2.5, 3.5)	-
**Number of patients with 50% or greater decrease in VAS**	**LBP**	n (%)	-	18 (15.0)	34 (28.3)	52 (43.3)	64 (53.3)	66 (55)	-
	**Leg pain**	n (%)	-	23 (19.2)	40 (33.3)	51 (42.5)	65 (54.2)	67 (55.8)	-
	**Main pain**^a^	n (%)	-	30 (25.0)	54 (45.0)	67 (55.8)	78 (65)	80 (66.7)	-
**ODI**		mean±SD	41.3±12.3	35±11.8	31.8±12.1	29.6±13.2	26.3±13.2	23.6±13.6	23.1±14.7
		diff	-	6.3 (4.2, 8.4)	9.5 (7.1, 12)	11.8 (9, 14.5)	15 (12.3, 17.7)	17.7 (14.8, 20.5)	18.3 (15.2, 21.3)
**SF-36**	**PF**	mean±SD	47.1±17.5	53.3±16	56.4±16.5	58.3±17.9	63.6±18.1	66.2±18.3	-
		diff	-	6.2 (3.3, 9.1)	9.3 (6.1, 12.5)	11.2 (7.7, 14.8)	16.5 (12.8, 20.2)	19.1 (15.3, 23)	-
	**RP**	mean±SD	38.5±22.2	48.8±21.4	55.2±23	58±24.3	61.4±24.2	65±25.6	-
		diff	-	10.3 (6.4, 14.1)	16.7 (12.7, 20.7)	19.5 (15.1, 24)	22.9 (18.3, 27.5)	26.5 (21.8, 31.2)	-
	**BP**	mean±SD	28.7±12.8	39.9±13.3	44.5±17.1	50.5±17.5	52.6±17.4	55.5±20.2	-
		diff	-	11.2 (8.7, 13.7)	15.8 (12.6, 19)	21.9 (18.2, 25.5)	24 (20.5, 27.5)	26.8 (22.9, 30.7)	-
	**GH**	mean±SD	41.4±16.8	45.1±16.5	46.9±18.9	48.6±17.5	49.6±18	51.1±19.6	-
		diff	-	3.8 (1.5, 6.1)	5.5 (3.1, 8)	7.2 (4.9, 9.5)	8.2 (5.7, 10.7)	9.8 (6.7, 12.8)	-
	**VT**	mean±SD	36.2±22.1	43.4±19.2	45.9±20.3	46.4±20.6	51.1±19.3	53.5±19.1	-
		diff	-	7.2 (3.5, 10.8)	9.7 (6.2, 13.3)	10.2 (6.4, 13.9)	14.9 (11.1, 18.8)	17.3 (13.6, 21.1)	-
	**SF**	mean±SD	48.1±21.1	59.6±21.8	64.3±21.2	65.6±21.8	69.5±20.5	71.8±20.7	-
		diff	-	11.5 (7.8, 15.1)	16.1 (12, 20.3)	17.5 (13.3, 21.7)	21.4 (17.1, 25.6)	23.6 (19.5, 27.8)	-
	**RE**	mean±SD	48.2±31.1	59±26.8	60.7±27.1	65±27.3	66.7±25.3	70.6±25.6	-
		diff	-	10.8 (5.7, 15.8)	12.5 (7, 18)	16.8 (11.1, 22.5)	18.5 (13, 23.9)	22.4 (16.7, 28.1)	-
	**MH**	mean±SD	54.3±21.7	61.2±20.3	62.6±19	63.3±20	66.2±19.3	68.5±17.6	-
		diff	-	6.8 (3.7, 10)	8.2 (5, 11.5)	9 (5.3, 12.7)	11.8 (8.3, 15.3)	14.2 (10.6, 17.9)	-
	**PCS**	mean±SD	38.4±12.7	46.1±12.7	49.8±15.1	52.3±15.3	55.7±15.3	58.3±16.8	-
		diff	-	7.7 (5.8, 9.7)	11.4 (9.1, 13.7)	14 (11.4, 16.5)	17.3 (14.6, 20)	19.9 (17, 22.8)	-
	**MCS**	mean±SD	45.6±16.9	53.6±15.4	56.1±16.6	57.7±17	60.5±16.2	63±16.4	-
		diff	-	8 (5.5, 10.5)	10.4 (7.8, 13.1)	12 (9.2, 14.9)	14.9 (12, 17.8)	17.4 (14.3, 20.5)	-
	**Total**	mean±SD	42.8±14.5	51.3±14	54.5±15.7	56.9±16.2	60±15.8	62.7±16.8	-
		diff	-	8.5 (6.3, 10.6)	11.7 (9.2, 14.2)	14.1 (11.3, 16.9)	17.2 (14.4, 20)	19.9 (16.9, 22.9)	-

Diff: Mean change (95% CI) compared with baseline

* As the 48 week follow-up was conducted by phone, pain level was assessed with numeric rating scale (NRS)

^a^ Area with greater pain out of low back and leg at baseline

ITT: intention-to-treat; VAS: Visual analog scale; LBP: low back pain; SD: standard deviation; ODI: Oswestry Disability Index; SF-36: Short-Form 36, PF: Physical functioning, RP: Role-physical, BP: Bodily pain, GH: General health, VT: Vitality, SF: Social functioning, RE: Role-emotional, MH: Mental health, PCS: Physical Component Summary, MCS: Mental Component Summary, CI: confidence interval

Analysis of medical use type and frequency showed that while 67 patients (55.8%) had taken analgesics during the 2 months prior to enrollment, only a few patients continued to take analgesics after the 16 week period. Also, while 25 patients (20.8%) had received 1 or more ESIs during the 2 month observational period prior to initiation of integrative Korean medicine treatment, few patients underwent ESI after completion of treatment despite the fact that the observational period was longer. Three patients were reoperated on within 1 year of study participation. An average 17 integrative Korean medicine treatment sessions were attended during the interventional period (16 weeks), and most patients received integrative Korean medicine treatment complying to the protocol with the exception of bee venom pharmacopuncture. Bee venom pharmacopuncture was replaced with an alternative pharmacopuncture solution in 57 patients (47.5%) based on skin test results and patient condition. Some participants continued to receive Korean medicine treatment after completion of treatment (Table 3).

**Table 3:** Medical use type and frequency by period.

Timepoint (period)	2 months previous to baseline (8 weeks)		Baseline to treatment completion (16 weeks)		Treatment completion to 24 week follow-up (8 weeks)		24 week follow-up to 1 year follow-up (28 weeks)	
	n (%)	mean±SD^a^(4 week average)	n (%)	mean±SD^a^(4 week average)	n (%)	mean±SD^a^(4 week average)	n (%)	mean±SD^a^(4 week average)
**Total number of subjects**	120		120		96		102	
**Additional surgery**					1 (1.0)		2 (2.0)	
**Analgesics (n)**	67 (55.8)	42.5±35.1 (21.3)*	27 (22.5)^d^	18.4±20.7 (4.6)	5 (5.2)	34.2±49.3 (17.1)	9 (8.8)	134.1±119.9 (19.2)
**Number of analgesic injections**					2 (2.1)	3.5±3.5 (1.8)	3 (2.9)	18.3±16.6 (2.6)
**Number of ESIs**								
**1 session**	13 (10.8)		2 (1.7)		2 (2.1)		5 (4.9)	
**2 sessions**	6 (5.0)						1 (1.0)	
**3 or more**	6 (5.0)							
**Number of physical therapy sessions**			1 (0.8)	1.0±0.0 (0.3)	2 (2.1)	3.0±0.0 (1.5)	7 (6.9)	24.8±26.0 (3.5)
**Number of exercise therapy sessions**			2 (1.7)	4.0±0.0 (1.0)	2 (2.1)	3.5±0.7 (1.8)	3 (2.9)	10.3±11.8 (1.5)
**Number of Korean medicine treatment sessions**^c^					41 (42.7)	6.5±4.2 (3.3)	31 (30.4)	11.5±14.0 (1.6)
**Acupuncture**		Not recorded	120 (100)^b^	17.0±4.8 (4.2)		Not recorded		Not recorded
**Electroacupuncture**		Not recorded	63 (52.5)^b^	17.2±6.1 (4.3)		Not recorded		Not recorded
**Pharmacoacupuncture**		Not recorded	120 (100)^b^	17.0±4.8 (4.2)		Not recorded		Not recorded
**Bee venom pharmacopuncture**		Not recorded	90 (75.0)^b^	15.4±5.8 (3.9)		Not recorded		Not recorded
**Other pharmacopuncture types**		Not recorded	57 (47.5)	10.8±7.9 (2.7)		Not recorded		Not recorded
**Chuna manipulation**		Not recorded	117 (97.5)^b^	16.1±4.8 (4.0)		Not recorded		Not recorded
**Herbal medicine (intake number)**		Not recorded	120 (100)^b^	191.4±43.6 (47.9)	29 (30.2)	82.3±37.0 (41.2)	18 (17.6)	59.9±62.3 (8.6)

* mean±SD value of 49 subjects. Subjects who had taken analgesics but were unsure of total intake amount were excluded in calculation

^a^ Total number of treatment sessions during this period (8 weeks, 16 weeks, 24 weeks, and 28 weeks post-baseline)

^b^ By predefined protocol (16 weeks of integrative Korean medicine treatment)

^c^ Total Korean medicine treatment use and frequency was assessed regardless of type

^d^ Subjects with rescue medicine intake

SD: standard deviation; ESI: epidural steroid injection

Pain and functional improvement was additionally analyzed by major pain characteristics. Between-group difference in VAS and ODI was non-significant in subgroups by onset of pain (relapse vs. continuation), and there were no time by group interactions either. Although between-group difference in VAS of LBP in subgroups of chronic pain (≥6 months) and non-chronic pain (<6 months) patients (p = 0.024) was significant, there were no time by group interactions. Meanwhile, ODI displayed time by group interactions (P<0.001), and patients with <6 months’ pain showed swifter initial functional improvement compared to chronic patients. Patients with main pain of LBP and those with main pain of leg pain showed time by group interactions in VAS of LBP and VAS of leg pain, respectively (P = 0.001, P<0.001), and both main pain sites exhibited swifter pain recovery compared to lesser pain sites. Difference by sex was observed in VAS of LBP and ODI results with men showing greater improvement compared to women (p = 0.003 and p = 0.012, respectively), but time by group interactions did not exist (Table 4).

**Table 4:** Results of subgroup analysis using repeated measures two-factor analysis of variance.

Category	n	Score	Group difference		Time by group interactions	
			F	p-value	F	p-value
Pain relapse vs. Continuation^a^	Continuation (79) Relapse (41)	LBP VAS	0.111	0.740	1.240	0.295
		Leg pain VAS	0.037	0.848	1.366	0.243
		ODI	2.062	0.154	1.341	0.245
≥6 months vs. <6 months^b^	<6 mons (33) ≥6 mons (87)	LBP VAS	5.233	0.024	0.543	0.743
		Leg pain VAS	1.243	0.267	2.276	0.052
		ODI	3.202	0.076	4.857	0.000
LBP or leg pain^c^	LBP (66) Leg pain (48)	LBP VAS	0.976	0.325	4.545	0.001
		Leg pain VAS	33.256	0.000	6.404	0.000
		ODI	1.819	0.180	0.945	0.466
Sex	Men (72) Women (48)	LBP VAS	9.100	0.003	0.580	0.715
		Leg pain VAS	2.949	0.089	1.273	0.280
		ODI	6.440	0.012	0.833	0.547

^a^ Pain relapse: Initial alleviation of pain after spine surgery and recurrence within 1 year; Continuation: Pain persisting after spine surgery

^b^ Pain duration period

^c^ Area with greater pain out of low back and leg at baseline. Excluded if low back pain = leg pain at baseline.

LBP: low back pain, VAS: Visual analog scale; ODI: Oswestry Disability Index

### Safety and adverse events in clinical observations

The greater proportion of adverse events with potential causal relationships with treatment was gastrointestinal-related. A total 32 patients reported one or more episodes of dyspepsia, diarrhea, or heartburn. Herbal medicine was reduced or temporarily suspended in 3 patients, and symptoms in the other patients subsided or were alleviated despite continuous administration. With the exception of 2 moderate adverse events, only mild adverse events were reported. The 2 moderate adverse events (LBP aggravation, and liver function abnormality in blood test) completely subsided within the follow-up period. Three patients showed mild erythema and urticaria, and all 3 recovered after bee venom pharmacopuncture was administered in reduced amounts and did not show additional allergic reactions.

Two patients showed increase of 2 times or over the upper limit of normal range in alanine aminotransferase (ALT), or a combined increase in total bilirubin (TB), aspartate aminotransferase (AST), and alkaline phosphatase (ALP), provided one of them was 2 times or over the upper limit of normal range in blood tests during the interventional period. Both patients were hepatitis B carriers, of which 1 patient was continuously administered herbal medicine without reduction in dosage and blood test results returned to normal at the next follow-up test, implying that there was no causal relationship with herbal medicine. The other patient concomitantly received orthopedic treatment for elbow pain along with herbal medicine after initiation of research, and in addition to repeatedly taking such medicines as chondroitin sodium sulfate, meloxicam, and etizolam, maintained alcohol intake of ≥half-bottle of soju (Korean distilled alcohol beverage) 2–3 times/week resulting in unclear causality of herbal medicine.

## Discussion

**Fig 2:**
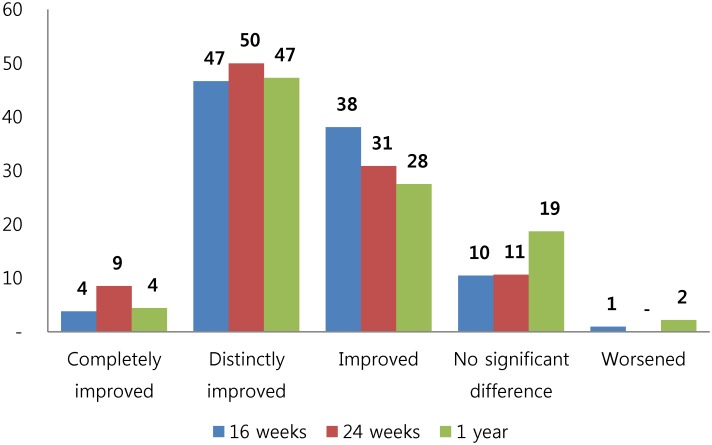
Satisfaction with current state (%).

The lumbar FBSS patients included in our study were chronic pain patients with moderate or higher pain levels, and displayed substantial improvement in LBP and leg pain, disability, and quality of life following 16 weeks of integrative Korean medicine treatment of which pain and disability maintained favorable state at 1 year follow-up. A total 89.4% of participants replied that their current state was improved at 6 months, and 79.2% at 1 year (Fig 2). Recently, several SCS studies of FBSS patients measured primary outcome as 50% or greater decrease in VAS of main pain site at 6 months [14–16,38], and 66.7% of patients displayed a 50% or greater decrease in main pain VAS at 6 months in this study. Also, although many patients had initially received conventional medicine (e.g. analgesics, ESI) prior to study participation, medication use decreased considerably after Korean medicine treatment and patients displayed preference for Korean medicine treatment. In subgroup analysis, patients with pain duration of <6 months displayed faster functional improvement, the region with greater pain out of low back and leg swifter pain alleviation, and men faster pain and functional improvement which may be partly explained through pain sensation difference between men and women [39].

The largest strength of this study is that it is a long-term prospective observation of results of integrative Korean medicine treatment in FBSS patients using various validated measurement instruments. Various effectiveness, safety, and adverse effect evaluation tools were used for comprehensive assessment of patient state. Medical use type and frequency were investigated in detail as observation of change in medical use patterns in these chronic pain patients before and after treatment may be of especial significance. The current treatment protocol closely resembles actual treatment patterns of Korean medicine hospitals in Korea and these study results should be informative to clinicians. The compliance rate for 16 weeks of intensive integrative Korean medicine treatment was high at 88%, and the number of patients presenting with adverse events of moderate or higher level requiring additional interventions was few.

There is a distinct paucity of research on Korean medicine treatment or CAM in FBSS patients. This is quite disproportionate considering the abundance of CAM research (e.g. acupuncture) relating to chronic LBP [40]. Two studies comparing SCS and conventional medical management (CMM) [16,40] included various multimodal treatments under the CMM umbrella and while acupuncture and manual therapy was applied in a small minority of patients, CAM was not the main method of treatment. Also, contrary to previous studies [16,41] reporting poor results for conservative treatment, the present study results of integrative Korean medicine treatment are favorable, and relevant hypotheses may be proposed drawing from previous research.

The effect of acupuncture on chronic LBP has been consistently described in previous literature [42], and there are several reviews reporting on the effects of bee venom pharmacopuncture on musculoskeletal pain [43]. Though research on Chuna manual therapy, a Korean manipulation method, is mostly found wanting, a 2013 systematic review tentatively summarized that it is effective for some musculoskeletal pains including LBP [29], and it is speculated to take effect by relaxing tight muscles and adjusting alignment in post-surgical pain.

The main herbal compound of the herbal medicine Chungpa-jun is also known as GCSB-5 (*Acanthopanax sessiliflorus*, *Achyranthes japonica*, *Saposhnikovia divaricata*, *Cibotium barometz*, *Glycine max*, and *Eucommia ulmoides*) and its anti-inflammatory [25], nerve regenerative [26], and cartilage protective effects [27] have been presented under experimental conditions, leading to its development and marketing as the natural product-derived drug ‘Shinbaro capsule’ in Korea. Chungpa-jun has also been shown to be effective for lumbar disc herniation in an observational study [20]. Many FBSS patients suffer digestive problems from long-term intake of analgesics, and this is shown in medication history of the current study also. For example, while 55.7% of patients took analgesics at baseline, the remaining patients accounted that they were unable to take analgesics notwithstanding their pain from indigestion problems. The non-inferiority of efficacy of GCSB-5 compared to Celecoxib in arthritis patients has been reported [28] along with fewer side effects including gastrointestinal problems. Similarly, the herbal medicine used in this study generally caused only mild gastrointestinal issues necessitating few dose reductions and allowing for relatively long and continuous intake.

While it is difficult to gauge which individual Korean medicine treatments are more important and effective for FBSS patients from the current study, they may be indirectly surmised from the results of a previous survey study conducted in spine-specialty Korean medicine hospitals in Korea [19]. KMD participants were asked to grade the importance of individual treatment methods for lumbar intervertebral disc herniation out of 7 levels (Importance: 1 = not important at all, 2 = unimportant, 3 = somewhat unimportant, 4 = neither important nor unimportant, 5 = somewhat important, 6 = important, and 7 = very important). Regarding short-term (8-week) treatment effect, bee venom (6.2) was rated to be most important, followed closely by pharmacopuncture (6.1) and acupuncture (6.1), while in the long-term (1-year), herbal medicine (6.5) received the highest score, with acupuncture, pharmacopuncture, and Chuna manipulation tying in second place (5.6). Though FBSS patients and intervertebral disc herniation patients are not within the same scope, it can be carefully suggested based on these results that invasive treatments such as bee venom are considered effective for controlling refractory pain similar to that seen in FBSS patients, and bee venom may be used in conjunction with other treatments such as pharmacopuncture and acupuncture for their relative safety as participants reported that bee venom was more frequently associated with risk of adverse reaction. In addition, functional recovery together with pain relief holds heightened significance in FBSS patients from a long-term perspective, to which aim herbal medicine was administered.

The biggest limitation of this study owes to its observational design of a single group lacking control, which design holds limited value as its evidence level is not high. Also, as integrative treatment was administered multimodally, the individual effects of treatments cannot be discerned. In addition, external validity cannot be ensured outside of Korea as the present outcomes reflect exposure to Korean medicine treatment in Koreans. While previous reports of integrative multimodal treatment outcomes (e.g. medication, injection, physical therapy, cognitive treatment) using CMM were generally negative compared to this study [16,41], whether the results of integrative Korean medicine treatment are superior is left to be verified in future rigorous randomized controlled trials (RCTs).

Despite these limitations, this study is the first long-term study to prospectively observe results of integrative Korean medicine treatment in FBSS patients. In conclusion, integrative Korean medicine treatment produced favorable results in various aspects including pain, function, and quality of life in chronic FBSS patients who generally do not respond well to CMM, and the results were maintained in the long term. However, these observational results of integrative Korean medicine treatment in FBSS patients need further verification through carefully designed RCTs of appropriate samples. Also, whether this method of treatment is cost-effective compared to other surgical or non-surgical treatments is a task left for future studies.
